# Cadmium Telluride Quantum Dots (CdTe-QDs) and Enhanced Ultraviolet-B (UV-B) Radiation Trigger Antioxidant Enzyme Metabolism and Programmed Cell Death in Wheat Seedlings

**DOI:** 10.1371/journal.pone.0110400

**Published:** 2014-10-20

**Authors:** Huize Chen, Yan Gong, Rong Han

**Affiliations:** 1 Higher Education Key Laboratory of Plant Molecular and Environmental Stress Response (Shanxi Normal University) in Shanxi Province, Linfen, China; 2 School of Life Science, Shanxi Normal University, Linfen, China; 3 School of Chemistry and Material Science, Shanxi Normal University, Linfen, China; Institute of Genetics and Developmental Biology, Chinese Academy of Sciences, China

## Abstract

Nanoparticles (NPs) are becoming increasingly widespread in the environment. Free cadmium ions released from commonly used NPs under ultraviolet-B (UV-B) radiation are potentially toxic to living organisms. With increasing levels of UV-B radiation at the Earth’s surface due to the depletion of the ozone layer, the potential additive effect of NPs and UV-B radiation on plants is of concern. In this study, we investigated the synergistic effect of CdTe quantum dots (CdTe-QDs), a common form of NP, and UV-B radiation on wheat seedlings. Graded doses of CdTe-QDs and UV-B radiation were tested, either alone or in combination, based on physical characteristics of 5-day-old seedlings. Treatments of wheat seedlings with either CdTe-QDs (200 mg/L) or UV-B radiation (10 KJ/m^2^/d) induced the activation of wheat antioxidant enzymes. CdTe-QDs accumulation in plant root cells resulted in programmed cell death as detected by DNA laddering. CdTe-QDs and UV-B radiation inhibited root and shoot growth, respectively. Additive inhibitory effects were observed in the combined treatment group. This research described the effects of UV-B and CdTe-QDs on plant growth. Furthermore, the finding that CdTe-QDs accumulate during the life cycle of plants highlights the need for sustained assessments of these interactions.

## Introduction

Quantum dots are semiconductor nanoparticles (NPs) that are increasingly used in industrial and biological applications [1]–[5]. The considerable amount of QDs released into the ecosystem raised concern due to their possible toxic effects in living organisms, particularly crop plants [6]–[8].

Cadmium telluride QDs (CdTe-QDs) are the most frequently used QDs [9]–[13]. However, the toxicity of CdTe-QDs is of increasing concern due to their small size (2–10 nm) and heavy metal formulation [14]. The cytotoxicity of CdTe-QDs was shown to be associated with their concentration and the duration of exposure [15]. CdTe-QDs release free cadmium ions (Cd^2+^) under UV-B irradiation, leading to the disruption of DNA replication, the displacement of Zn^2+^ in Zn finger structures, and the generation of reactive oxygen species (ROS) [16], [17]. Furthermore, the toxicity of CdTe-QDs has been shown to be much higher than that of Cd^2+^ [12] and that the cytotoxicity was not solely due to free Cd^2+^ [13]. Cellular toxicity was found to be closely related to the size of quantum dots, with smaller diameter QDs (2 nm) demonstrating more toxic effects than larger QDs (5 nm) [18]. These studies demonstrated the potential toxic effects of QDs on inducing antioxidative enzyme activity and DNA replication processes in plants.

Owing to their unique sedentary lifestyle, plants are predicted to be most affected by the accumulation of CdTe-QDs in the environment. This effect is likely to be further exacerbated by the increasing levels of solar UV-B radiation (290–320 nm) at the Earth’s surface due to the depletion of stratospheric ozone. Current levels of UV-B during the cropping season are somewhere between 2–12 kJ/m^2^/d on the Earth’s surface, which exhibits an increase of 6–14% of UV-B radiation over the pre-1980 levels [19]. Based on the UV-B dose per 10 min, the computative calculation of the erythermal UV-B doses range from 2 kJ/m^2^ to 9 kJ/m^2^ on a clear-sky day on 31 August 2014 (themis.nl). It has been estimated that a 1% decrease in ozone will lead to an increase in UV-B for about 2%. GISS (Goddard Institute for Space Studies) modeling showed a springtime enhancement of erythemal UV doses of up to 14% in the Northern hemisphere and 40% in the Southern hemisphere [20]. Plants are continuously exposed to solar UV-B radiation as a result of their sessile lifestyle [21]. UV-B has the highest energy of any part of the daylight spectrum and has the potential to damage macromolecules including DNA [22], generate ROS, and impair cellular processes [23]–[25]. Recently, UV-B radiation was found to act as a key environmental signal that initiated diverse responses in plants affecting metabolism, development and viability [26].

The impact of the environmental accumulation of CdTe-QDs in the presence of enhanced UV-B radiation on living organisms in particular plants is largely unknown. Further studies are needed to understand the interactions of plants, QDs, and enhanced UV-B radiation at the biochemical and molecular levels.

In this study, we investigated the individual and combined effects of QDs and UV-B radiation on an important crop, wheat. We assessed the effect of QDs and UV-B radiation, either alone or in combination, on seedling growth, the activation of antioxidant enzymes, the distribution of CdTe-QDs in root cells, and the induction of programmed cell death.

## Materials and Methods

### Synthesis of CdTe-QDs

Water-soluble CdTe-QDs were prepared as described [27]. Millipore water (100 mL) was degassed with argon for approximately 1 hour. Cd(ClO_4_)_2_.6H_2_O and 3-mercaptopropanoic acid were added and the pH adjusted to 11.3 with 1 M NaOH. The solution was gassed under argon for additional 30 min. H_2_Te gas, generated from Al_2_Te_3_ by adding 0.5 M H_2_SO_4_ dropwise, was bubbled under a slow argon flow for approximately 10 min. The solution was subsequently refluxed for 2 hours. Samples were vacuum dried and stored in darkness before use.

### QDs characterization

QDs were characterized using a JEM-2100 (JEOL, Japan) transmission electron microscope (TEM), and a D8 Advance (Bruker, Germany) X-ray diffractometer (Cu K_α_). Samples were prepared by drying sample droplets from water dispersion onto a 100-mesh Cu grid coated with a lacey carbon film, which was dried prior to imaging by TEM. Absorption and emission spectra were collected using fluorescence spectroscopy (JASCO FP-8000).

### Seedling cultivation and chlorophyll content

Wheat seeds (*Triticum aestivum* L. *cv.* JIN-8) were sterilised for 10 min with 1% NaClO and then washed for 10 min in running distilled water. Thirty seeds were cultured per Petri dish in a growth chamber at 25°C with 60% relative humidity and were watered daily. Four treatments, each with three replicates, were applied, starting on the day of seed germination as outlined in Table 1. Briefly, seeds were soaked with either QDs [8] or water for the control group all day long. UV-B radiation was applied 8 h/day during the light cycle. Five graded doses of UV-B radiation intensities were tested: (B1) 2.5 KJ/m^2^/d; (B2) 5 KJ/m^2^/d; (B3) 7.5 KJ/m^2^/d; (B4) 10 KJ/m^2^/d and (B5) 12.5 J/m^2^/d. CdTe-QDs were applied at five different concentrations: (C1) 25 mg/L; (C2) 50 mg/L; (C3) 100 mg/L; (C4) 200 mg/L and (C5) 400 mg/L. The effect of UV-B and QDs doses on seedling growth were determined by assessing height, root length, and the concentrations of malondialdehyde (MDA), soluble sugar and soluble protein. Five-day-old wheat seedlings were subsequently treated with the doses showing the most inhibitory effect and more detailed analyse including plant height, root length, biomass, and chlorophyll content were performed. After 5 d of growth, the plant height and root length were measured with a ruler. Twenty seedlings per replicate per treatment were randomly selected for analysis. A total of 90 seedlings were assessed for height, fresh weight (FW), and dry weight (DW). Fresh and dry weights were detected with an analytical balance. Chlorophyll content was measured as described [28].

**Table 1:** Light/dark cycles and treatment regimens with UV-B and CdTe-QDs.

Group	Treatment	Light	UV-B irradiation	Dark	CdTe-QDs treatment
CK	Control	8 h/d	-	16 h/d	dH_2_O
B	UV-B	8 h/d	8 h/d	16 h/d	dH_2_O
C	CdTe-QDs	8 h/d	-	16 h/d	CdTe-QDs solution
B+C	UV-B+CdTe-QDs	8 h/d	8 h/d	16 h/d	CdTe-QDs solution

### Measurement of malondialdehyde (MDA), soluble sugar, and soluble protein concentrations

MDA concentration was determined using the trichloroacetic acid (TCA) method. Fresh tissues (1.0 g) were ground with SiO_2_ in 2 mL 10% TCA. After centrifugation at 4000 rpm for 10 min, supernatants were removed, added to 2 mL 0.6% (w/v) thiobarbituric acid, and incubated in a 100°C water bath for 15 min. Reactions were stopped on ice. After centrifugation at 4000 rpm for 15 min, supernatants were assayed at 532 and 450 nm.

Total sugar concentration was determined using anthrone colorimetry. Dry plant tissues (50 mg) were triturated with 4 mL 80% ethyl alcohol. Supernatants were collected after 40 min continuous stirring in a water bath at 80°C. Activated carbon (10 mg) was used to decolorize the solution for 30 min, after which 5 mL anthrone were added and samples were incubated in a water bath at 100°C for 10 min. Samples were then cooled for 5 min before spectrophometric absorbance assessment at 625 nm. Concentrations were determined using standard curves.

Soluble protein was extracted according to Zhao [29], using bovine serum albumin as a calibration standard. Fresh tissues (1.0 g) were ground in 6 mL distilled water and then centrifuged at 4000 rpm for 15 min. After centrifugation, supernatants were added to 5 mL Coomassie brilliant blue G-250 and incubated at room temperature for 15 min before spectroscopic assessment at 595 nm.

### Cd^2+^ Accumulation

The concentration of Cd^2+^ within shoots and roots was analyzed using Inductively Coupled Plasma Mass Spectrometry (ICP-MS) [30]. After exposure to CdTe-QDs for 5 days, 4–7 plants were recovered and roots were rinsed thoroughly with deionized water to remove material that was neither adsorbed nor integrated into the plant tissues. Roots and leaves were separated, oven dried (70°C, 24 h) and weighed. Dried tissues were digested in 4∶1 concentrated HNO_3∶_30% H_2_O_2_ for at least 2 h on a hotplate at 60°C. Samples were transferred to polypropylene tubes and centrifuged at 1000 g for 10 min. Supernatants were recovered and brought to 10 mL using deionized water. Cd analysis was performed using an X-Series ICP-MS instrument with an ICP-MS elements solution set used as standards.

### Antioxidant Assays

Roots and shoots were separately homogenized, centrifuged and extracts assayed for catalase (CAT), guaiacol peroxidase (GPOX), superoxide dismutase (SOD), ascorbate peroxidase (APOX), dehydroascorbate reductase (DHAR) and glutathione reductase (GR) activities as previously described [31]–[35].

### Measurement of ROS Production

Determination of H_2_O_2_ content in plant extracts. Frozen plant tissues (0.1 g) were homogenized in a 1∶9 (w/v) phosphate buffer (50 mM, pH 6.0) at 4°C. The content of H_2_O_2_ was analyzed with a hydrogen peroxide assay kit (Beyotime, China) according to the manufacturer instruction. Briefly, test tubes containing 50 µL test solutions were placed at room temperature for 30 min and measured immediately with a spectrometer at a wavelength of 560 nm. Absorbance values were calibrated to a standard curve generated with known concentrations of H_2_O_2_ [36]. O_2_^−^ levels were monitored by staining for 20 min in a solution of 2 mM NBT in 20 mM phosphate buffer (pH 6.1). The reaction was stopped by transferring the seedlings into distilled water. O_2_^−^ content was quantified using the method of Ramel et al. [37]. The NBT-stained plantlets were ground in liquid nitrogen; the obtained powder was solubilized in 2 M KOH-dimethyl sulfoxide (1∶1.16, v/v) and then centrifuged for 10 min at 12000 g. The A630 was immediately measured and compared with a standard curve obtained from known amounts of NBT in the KOH-dimethyl sulfoxide mixture [38].

### Confocal Fluorescence Imaging Analysis

Cellular fluorescence was monitored using a FV-1000 Confocal system (Olympus, Co.) with a 1.4 NA, 60×oil immersion objective lens. Cell observations were performed on at least three replicate samples. Quantum dots were excited at 488 nm. Nuclei were stained with 4′,6-diamidino-2-phenylindole (DAPI) (Sigma, USA) and excited at 405 nm.

### DNA laddering

Genomic DNA was extracted using a standard CTAB protocol [39]. DNA concentration was determined by spectrophotometry. DNA (5 µg) was analyzed using 1.5% agarose gel electrophoresis in the presence of ethidium bromide (5 µg/mL) and gels were visualized on a UV transilluminator.

### Statistical Analysis

Data results are expressed as means ± standard deviations (SD). Statistical significance was assessed using one-way analysis of variance (ANOVA) tests using General Linear Model followed by Tukey test was performed using the SPSS 21.0 and Sigma-plot 10.0.

## Results and Discussion

### QDs characteristics

**Figure 1:**
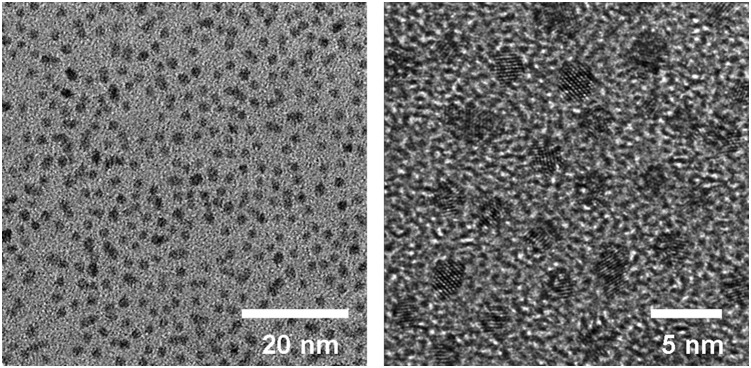
TEM image of synthesized CdTe-QDs.

**Figure 2:**
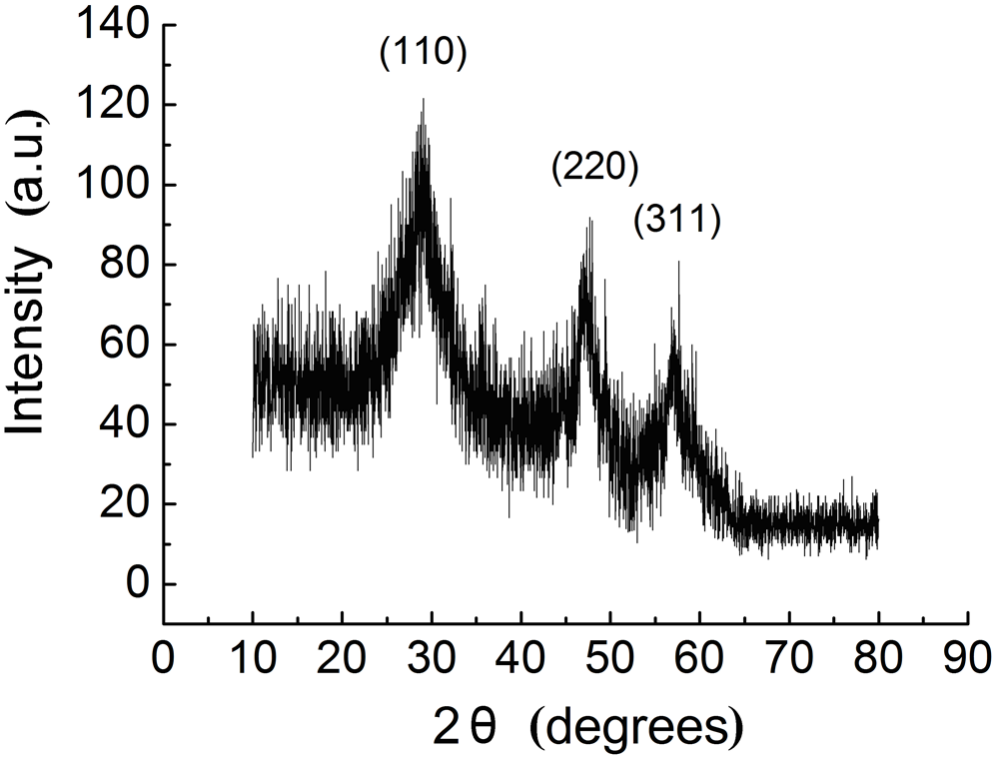
X-ray diffraction spectrum of CdTe-QDs.

**Figure 3:**
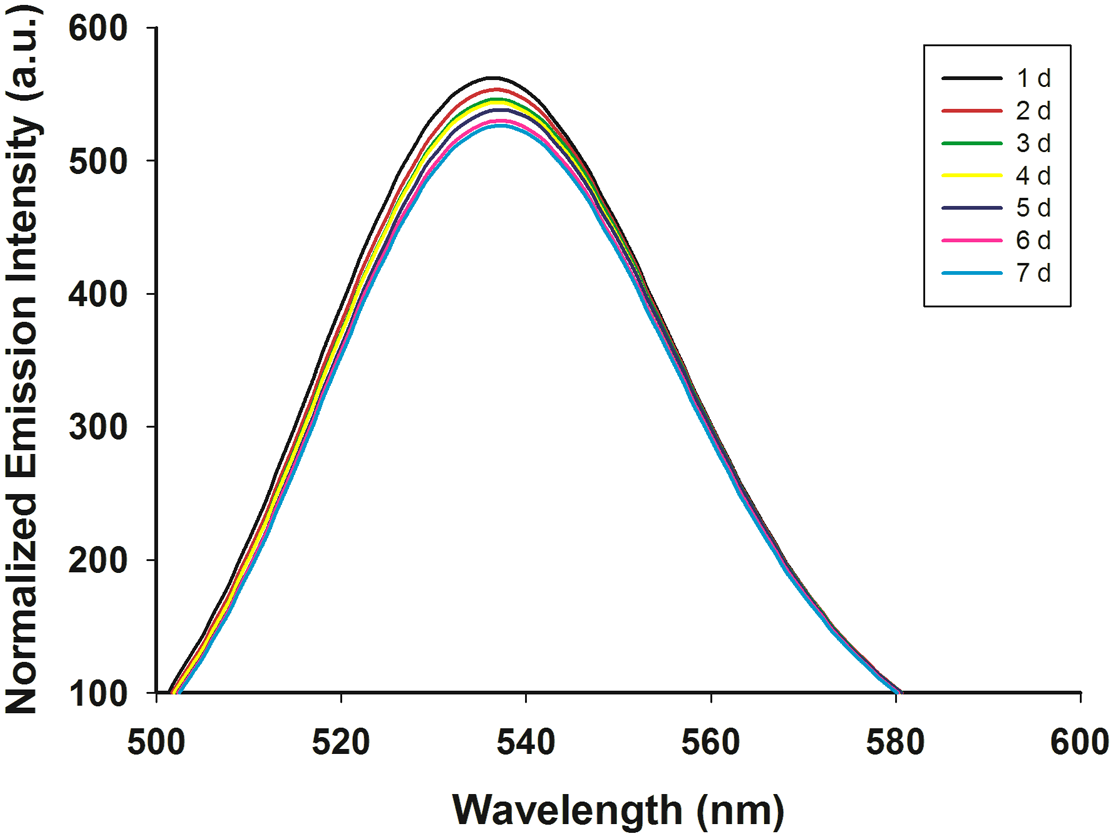
Emission spectra of QDs in H_2_O over 7 days.

CdTe-QDs were synthesized and characterized using TEM. CdTe-QDs had an average diameter of 2.3±0.2 nm (Figure 1). X-ray diffraction (XRD) spectra scanned over the 2 theta (θ) range of 10–80° showed a cubic XRD structure of CdTe-QDs with diffractive peaks of 28°, 46°, and 57°, indicating well-crystallized QDs (Figure 2). The QDs exhibited narrow fluorescence emission spectra (λ max = 531 nm). Emission spectra collected over a period of 7 days are shown in Figure 3. QDs emission intensity decreased daily, with a total decrease of approximately 6.5% over 7 days (normalized intensity change from 561.8488 to 525.1871). These results indicated that almost 93.5% of QDs persisted during the experimental exposure period.

### The effect of CdTe-QDs and UV-B irradiation on the growth of wheat seedlings

**Figure 4:**
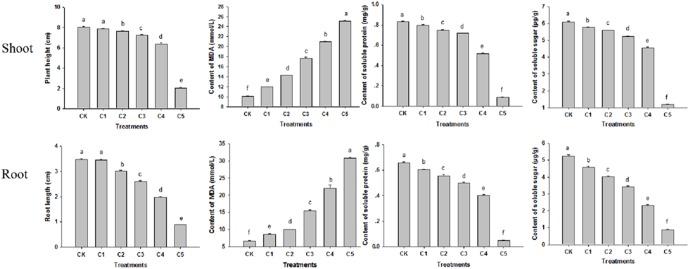
Effects of different CdTe-QDs concentrations on 5-day-old wheat seedlings. CK, control group; C, treatment groups with different concentrations of CdTe-QDs (C1, 25 mg/L; C2, 50 mg/L; C3, 100 mg/L; C4, 200 mg/L; C5, 400 mg/L). Data are means±SD (n = 3). Means with the same letter are not significantly different at Tukey’s test (p≤0.05).

**Figure 5:**
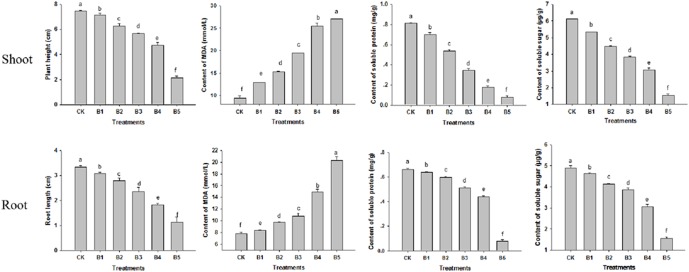
Effects of different enhanced UV-B radiation doses on 5-day-old wheat seedlings. CK, control group; B, treatment groups with graded doses of UV-B radiation (B1, 2.5 KJ/m^2^/d; B2, 5 KJ/m^2^/d; B3, 7.5 KJ/m^2^/d; B4, 10 KJ/m^2^/d; B5, 12.5 KJ/m^2^/d). Data are means±SD (n = 3). Means with the same letter are not significantly different at Tukey’s test (p≤0.05).

Wheat seedlings were treated with single agents of either CdTe-QDs or UV-B irradiation. Treatments of seedlings with five different concentrations of CdTe-QDs resulted in reductions in shoot height and root length, increased lipid oxidation as measured by the levels of the oxidation product MDA, as well as reductions in soluble sugar and soluble protein concentrations, in a dose dependent manner when compared with the control (CK) group (Figure 4). Of five CdTe-QDs concentrations tested, the concentration of 200 mg/L (C4) was selected for further evaluations when taking into account of all parameters assessed (almost 75% shoot/root length reduction and 15 times MDA increase performed in the C5 treatment) (Figure 4). Treatments of seedlings with graded doses of UV-B irradiation led to similar growth inhibition as assessed by the above parameters (Figure 5). The UV-B dose of 10.0 KJ/m^2^/d was selected for subsequent experiments since the dose higher than 10.0 KJ/m^2^/d strongly inhibited wheat growth (Figure 5). This dose of UV-B irradiation should decrease the density of O_3_ by 20% [40].

### CdTe-QD accumulation in plant tissues

**Figure 6:**
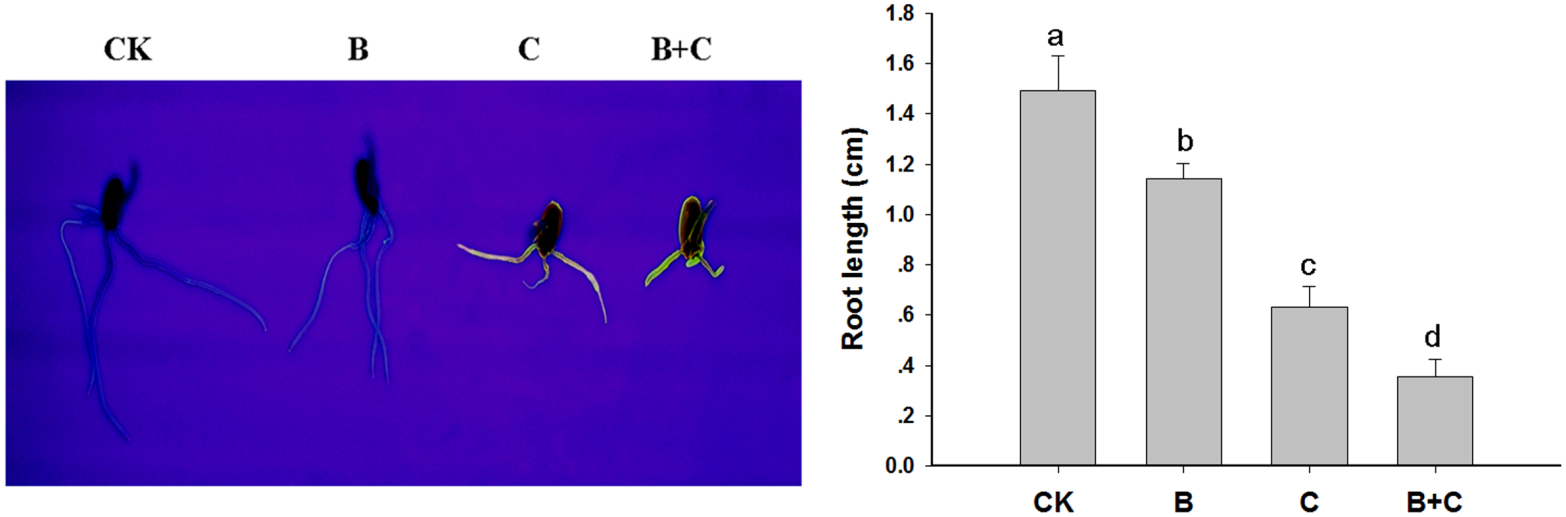
Two-day old seedlings visualized with UV light. Control, CK; UV-B treatment alone, B; CdTe-QDs treatment alone, C; combined CdTe-QDs and UV-B treatment, C+B.

Wheat seedlings were randomly assigned to four treatment groups: control (CK), UV-B at 10 KJ/m^2^/d (B), CdTe-QDs at 200 mg/L (C), and UV-B (10 KJ/m^2^/d) plus CdTe-QDs (200 mg/L) (B+C). The uptake of CdTe-QDs was evident in two-day-old treated seedlings in the group treated by CdTe-QDs alone (C) and that treated by UV-B (10 KJ/m^2^/d) plus CdTe-QDs (200 mg/L) (B+C) (Figure 6). Root length was reduced in all three treatment groups compared to the control group (Figure 6). CdTe-QDs fluorescence was observed in roots of seedlings treated with CdTe-QDs alone (C) and in seedlings received combined CdTe-QDs and UV-B (B+C), indicating CdTe-QDs nanoparticles uptake by roots.

**Figure 7:**
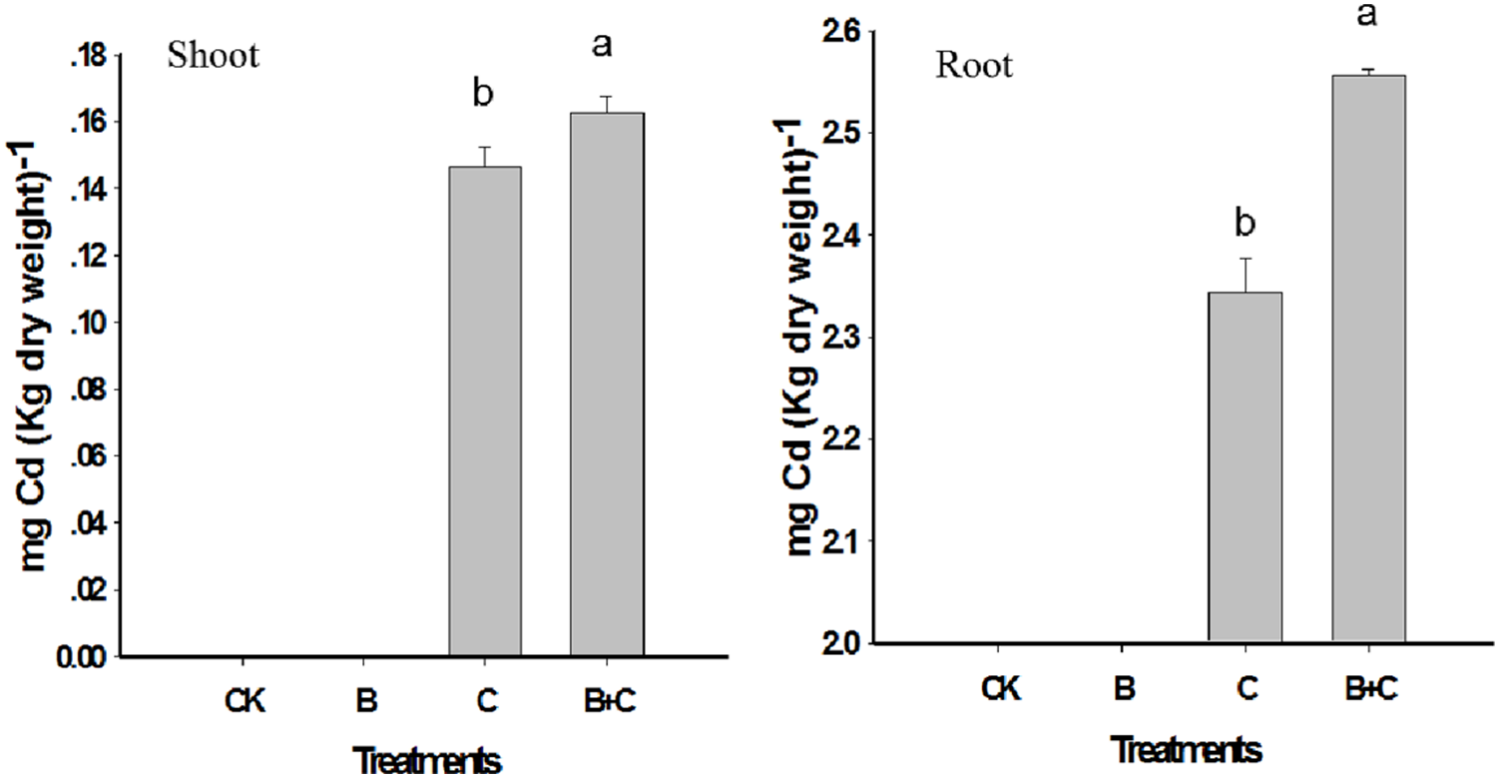
Cd concentration in tissues of 5-day-old wheat seedlings under different treatments. Control, CK; UV-B treatment alone, B; CdTe-QDs treatment alone, C; combined CdTe-QDs and UV-B treatment, C+B.

**Figure 8:**
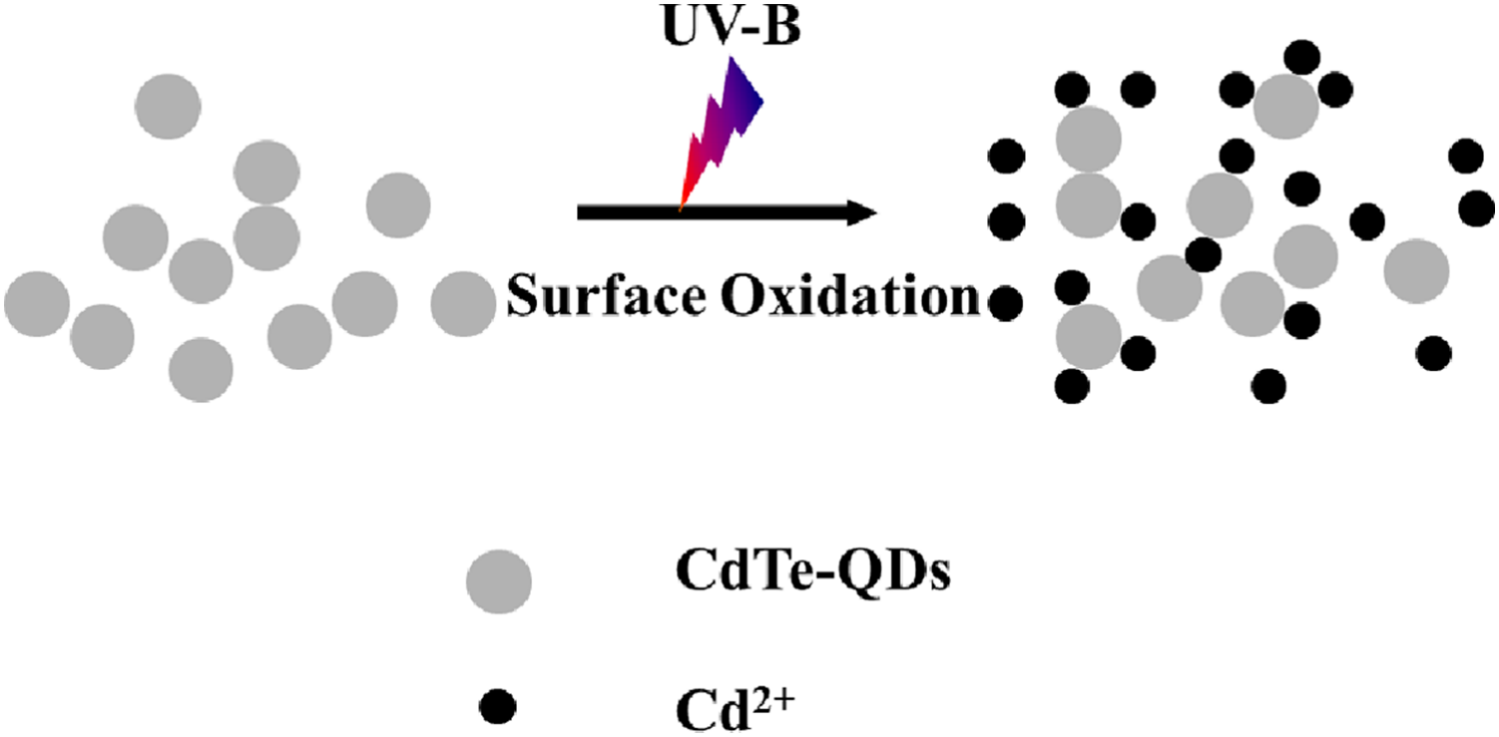
Schematic showing Cd^2+^ release from QDs as a result of UV-B oxidation of CdTe-QDs surfaces [41], [42].

Treatment of seedlings with CdTe-QDs resulted in a higher Cd accumulation in roots (2.344 mg/Kg DW) than in shoots (0.1461 mg/Kg DW) (Figure 7). This is likely due to the direct contact exposure of roots to the CdTe-QDs suspension. There was also a marked increase in Cd concentration in both shoots and roots of seedlings treated with CdTe-QDs in combination with UV-B irradiation (Figure 7). This increase in the levels of Cd^2+^ in the presence of UV-B radiation was reported previously as a consequence of QDs surface oxidation and Cd^2+^ release (Figure 8) [41], [42]. Our results suggest a potential additive effect of CdTe-QDs and UV-B on plants.

### Plant growth and chlorophyll content

The growth and chlorophyll contents of wheat seedlings exposed to different treatments were determined (Tables 2 and 3). UV-B radiation treatment resulted in a significantly reduction in plant height with an average height 30.1 mm shorter than that of controls (Table 2). Similarly, chlorophyll content, both chlorophyll *a* and *b*, were significantly lower than in controls (Table 3). The phytotoxic effects of CdTe-QDs treatment on plant height and chlorophyll content were less severe than those of UV-B radiation. However, root length was more severely affected in the CdTe-QDs group compared to the UV-B treated group (Table 2). Average root length in CdTe-QDs-treated plants was 14.74 mm shorter than in untreated controls. Total biomass was similar between the UV-B and QDs treated groups, but was significantly reduced compared to control untreated seedlings. The combined treatment group showed a significant reduction in plant height, root length, and total biomass when compared to the control group and the single treatment groups (Table 2). The enhanced toxicity in the combined treatment group may have been due to the release of free Cd^2+^ from CdTe-QDs in the presence of UV-B radiation.

**Table 2:** Effects of QDs and UV-B radiation on growth of 5-day-old wheat seedlings.

Group	Treatments	Plant height (mm)	Root length (mm)	Fresh weight (g)	Dry weight (g)
CK	Control	75.63±1.379a	26.87±0.493a	0.1983±0.003a	0.0307±0.002a
B	UV-B	45.53±1.193c	16.26±0.404b	0.1217±0.011c	0.0143±0.003c
C	CdTe-QDs	51.23±1.101b	12.13±0.493c	0.1393±0.007b	0.0187±0.003b
B+C	UV-B+CdTe-QDs	31.47±1.026d	7.96±0.776d	0.0980±0.003d	0.0080±0.001d

Data are means±SD (n = 3). Means with the same letter are not significantly different at Tukey’s test (p≤0.05).

**Table 3:** Chlorophyll content in tissues of 5-day-old wheat seedlings.

Group	Treatments	Chlorophyll *a* (mg/g FW)	Chlorophyll *b* (mg/g FW)	Total chlorophyll (mg/g FW)	Chlorophyll *a*/*b* ratio
CK	Control	0.5943±0.0068a	0.2723±0.0042a	0.8667±0.0059a	2.1828±0.0510a
B	UV-B	0.4030±0.0076c	0.1970±0.0056c	0.6000±0.0026c	2.0474±0.0949b
C	CdTe-QDs	0.4990±0.0061b	0.2473±0.0057b	0.7463±0.0012b	2.0186±0.0714b
B+C	UV-B+CdTe-QDs	0.3443±0.0047d	0.1763±0.0084d	0.5207±0.0038d	1.9564±0.1165b

Data are means±SD (n = 3). Means with the same letter are not significantly different at Tukey’s test (p≤0.05).

### Antioxidant defense in wheat seedlings

**Figure 9:**
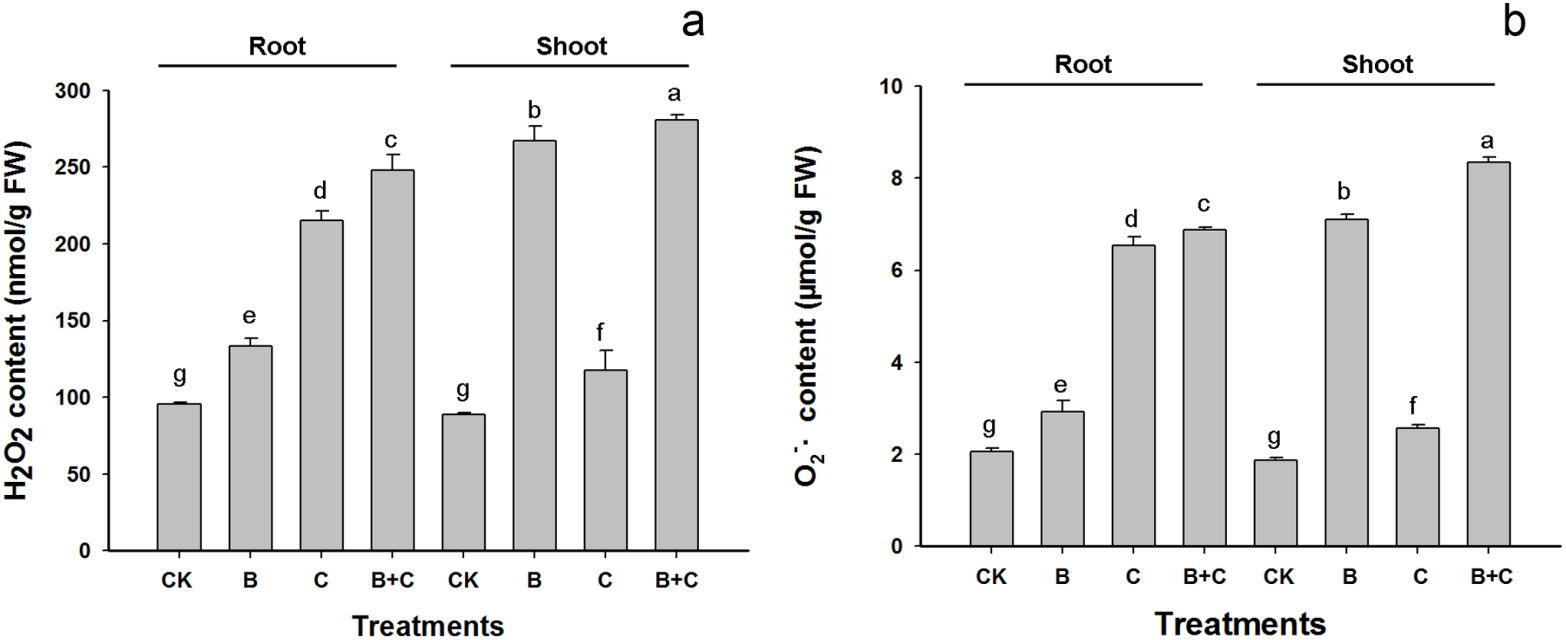
Contents of H_2_O_2_ and O_2_^−^ in the root and shoot of 5 day old seedlings following different treatments. Data are means±SD (n = 3). Means with the same letter are not significantly different at Tukey’s test (p≤0.05).

The cytotoxicity of QDs and UV-B radiaton have shown to be associated with the production of ROS [6], [43], [44]. H_2_O_2_ and O_2_^−^ are the most common ROS produced during normal cellular metabolic processes. Their imbalance can be highly cytotoxic [35], [45]. To determine the involvement of H_2_O_2_ and O_2_^−^ during QDs and UV-B exposure, 5-day-old seedlings were examined for the presence of ROS following treatments with CdTe-QDs, UV-B, and combined CdTe-QDs and UV-B. The results showed that there was a marked increase in the concentrations of H_2_O_2_ in roots treated with CdTe-QDs, either alone or in combination with UV-B, compared to the UV-B treated and untreated groups. Furthermore, there was 11.5% increase in the levels of H_2_O_2_, from 215.37 nmol/g FW in the CdTe-QD treated group to 247.89 nmol/g FW in the combined CdTe-QD and UV-B treated group (Figure 9). Similar results were observed for the O_2_^−^ level. These results are consistent with the greater toxicity from higher levels of Cd following UV-B irradiation predicted for roots. We noted that such additive effect by UV-B was moderate in shoots where the production of H_2_O_2_ and O_2_^−^ is found to be primarily associated with UV-B radiation. The relatively moderate effect in the Cd-associated ROS production in shoots is likely due to the low levels of Cd accumulation in shoots (Figure 7).

**Figure 10:**
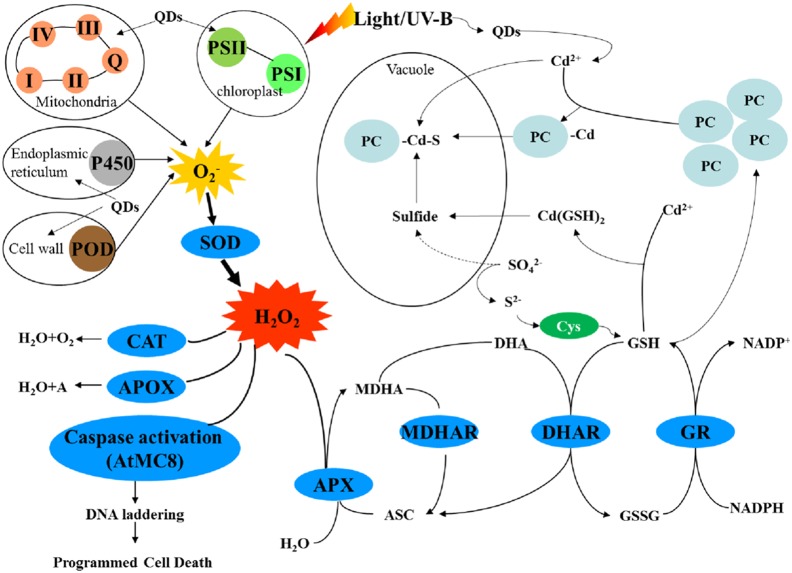
Schematic of the antioxidative defense system and Cd degradation mechanism in plants.

Plants use a highly complex antioxidant defense system to protect against oxidative stress that includes antioxidant enzymes such as SOD, CAT, GPOX, APOX, DHAR, and GR (Figure 10) [35], [46]. The O_2_^−^ generated from mitochondria, chloroplasts, endoplasmic reticulum, and the cell wall is reduced to H_2_O_2_ by SOD. H_2_O is then formed from H_2_O_2_ through the activities of CAT, and APOX. DHAR and glutathione reductase (GR) participate in the transformation of glutathion (GSH) to oxidized form of glutathione (GSSG) and generation of nicotinamide adenine dinucleotide phosphate (NADPH). GSH can bind to free Cd^2+^, forming Cd(GSH)_2_ and leading to the reduction of the Cd toxicity. In addition, phytochelatins (PCs) may bind Cd to form PC-Cd-S, which can then be stored in the vacuole [47]. Finally, H_2_O_2_ activation of caspase can lead to programmed cell death [44], [48].

**Figure 11:**
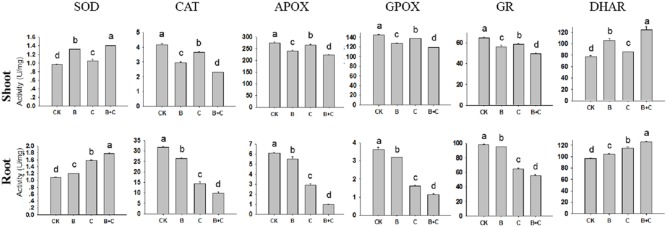
Antioxidant enzyme activities in shoots and roots of 5-day-old wheat seedlings. Data are means±SD (n = 3). Means with the same letter are not significantly different at Tukey’s test (p≤0.05).

Antioxidative defense enzymes activities were further assessed in the control, UV-B-only treated, CdTe-QDs treated, and UV-B plus CdTe-QDs treated seedlings (Figure 11). The inhibitory effect of CdTe-QDs upon antioxidant enzymes activities was milder than that of UV-B radiation treatment in shoots. Photosynthesis, which is susceptible to changes in light, mainly occurs in leaves [49]. Under ambient UV-B, the UV resistance locus 8 (UVR8) protein mediates antioxidant enzyme activities and regulates plant growth. However, it is currently unknown whether UV-B irradiation used in this study stimulates antioxidant enzyme activities in the same pathway.

In shoots, SOD and DHAR activities significantly increased in seedlings exposed to UV-B radiation when compared to controls. However, the other enzymes were significantly inhibited in the UV-B treated group compared to the control group. There were also significant difference in enzyme activities between the CdTe-QDs treated group and the control group, but the effects were not as marked as with the UV-B treated group (Figure 11 upper part). We inferred that CdTe-QDs affected metabolism in shoots through the release and transport of Cd^2+^ ions from QDs in root cells.

**Figure 12:**
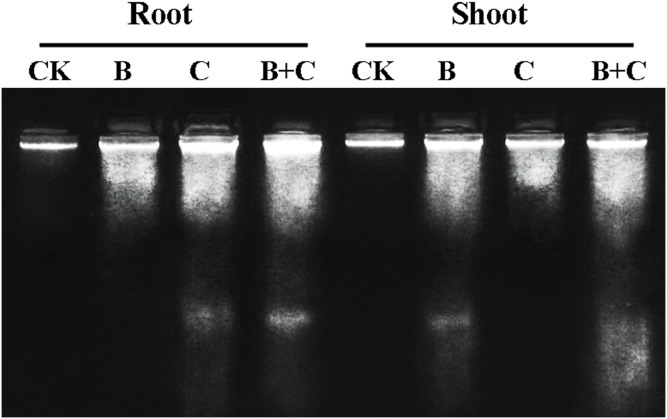
DNA laddering in wheat tissues. Electrophoresis gels of DNA extracted from wheat with different treatments. CK: control group; B: enhanced UV-B radiation; C: CdTe-QDs treatment; B+C: combined UV-B and CdTe-QDs treatment.

The cytotoxicity of Cd^2+^ has been known to be associated with oxidation stress in mammalian cells. We have demonstrated the additive effect of UV-B radiation on the Cd^2+^ toxicity in roots, manifested by the decreased levels of antioxidant enzymes such as CAT, APOX, GPOX and GR (Figure 11), and increased DNA fragmentation (Figure 12). The more pronounced effects in roots is presumably due to the release of additional Cd^2+^ as a result of surface oxidation of QDs under UV-B radiation [41], [42]. We inferred that cytoplasmic diffusion of free Cd^2+^ occurred more rapidly than the diffusion of NPs. The greater root toxicity observed with the combined treatment compared to the single CdTe-QDs treatment may be the consequence of the increased accumulation of intracellular Cd^2+^.

### Distribution of CdTe-QDs in root cells

**Figure 13:**
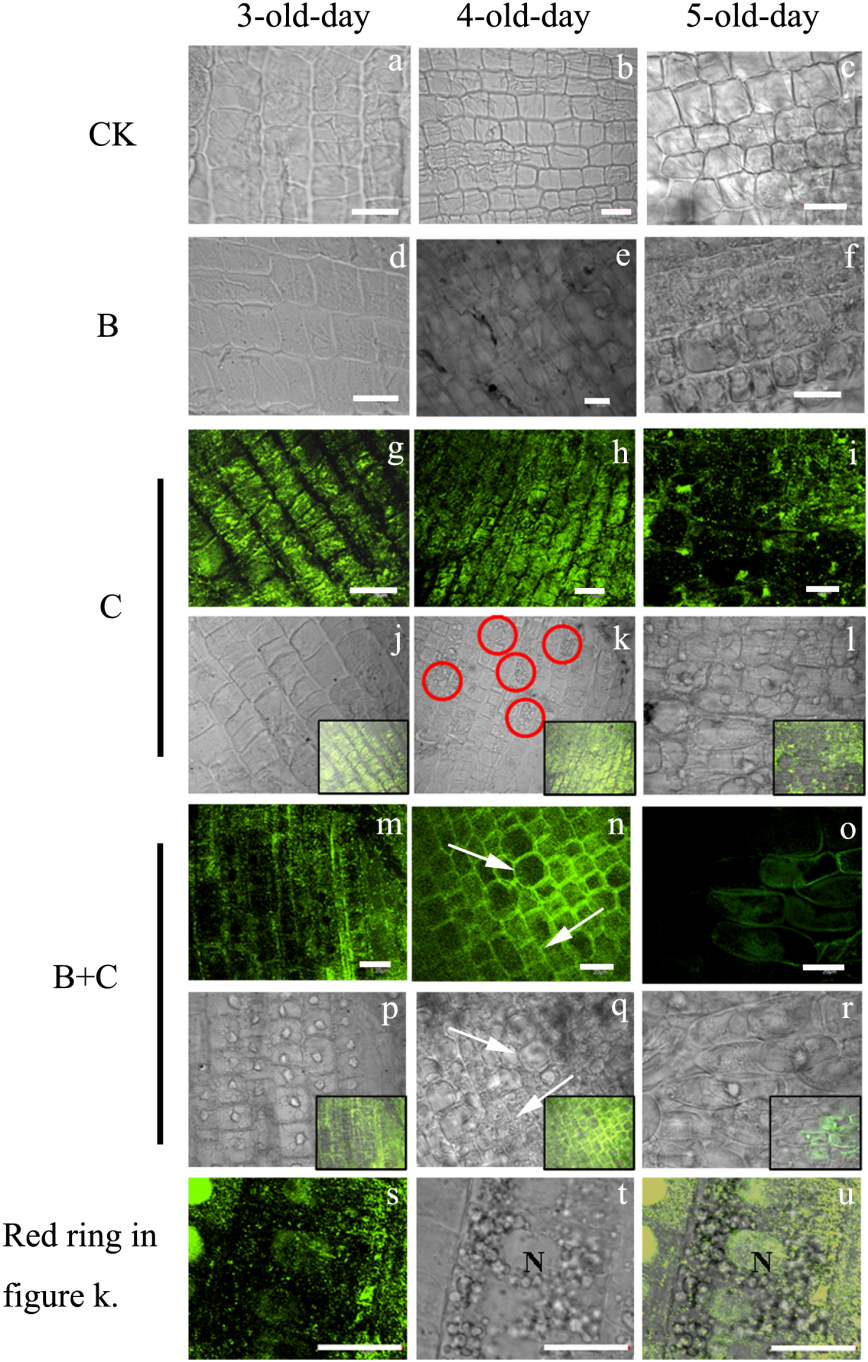
Confocal imaging of CdTe-QDs distribution in wheat root cells. CdTe-QDs are indicated by fluorescence imaging (g, h, i, m, n, o, s), DIC images demonstrate cell integrity (a, b, c, d, e, f, j, k, l, p, q, r, t). Regions highlighted in red in panel e are shown in panels s, t, and u. Pictures of the bottom right corner in figure j, k, l, p, q, r is the merge figures of g and j, h and k, i and l, m and p, n and q, o and r separately. Scale bars = 20 µm.

It has been demonstrated that CdSe-QDs, through the apoplastic pathway with the aid of silwet L-77, could be transferred into roots and were stable [50]. We therefore examined root cells of wheat seedling for the presence of CdSe-QDs using confocal microscopy (Figure 13). No fluorescence signal was detected in the untreated control (a, b, c) and in the UV-B treated cells (d, e, f). As shown in the DIC images, cells in the control group exhibited a clear outline of cell wall and regular shape ove three day period. In contrast, cells in the UV-B treated group showed the presence of intracellular vesicles on the fifth day and apparent abnormal rectangular shapes (f).

**Figure 14:**
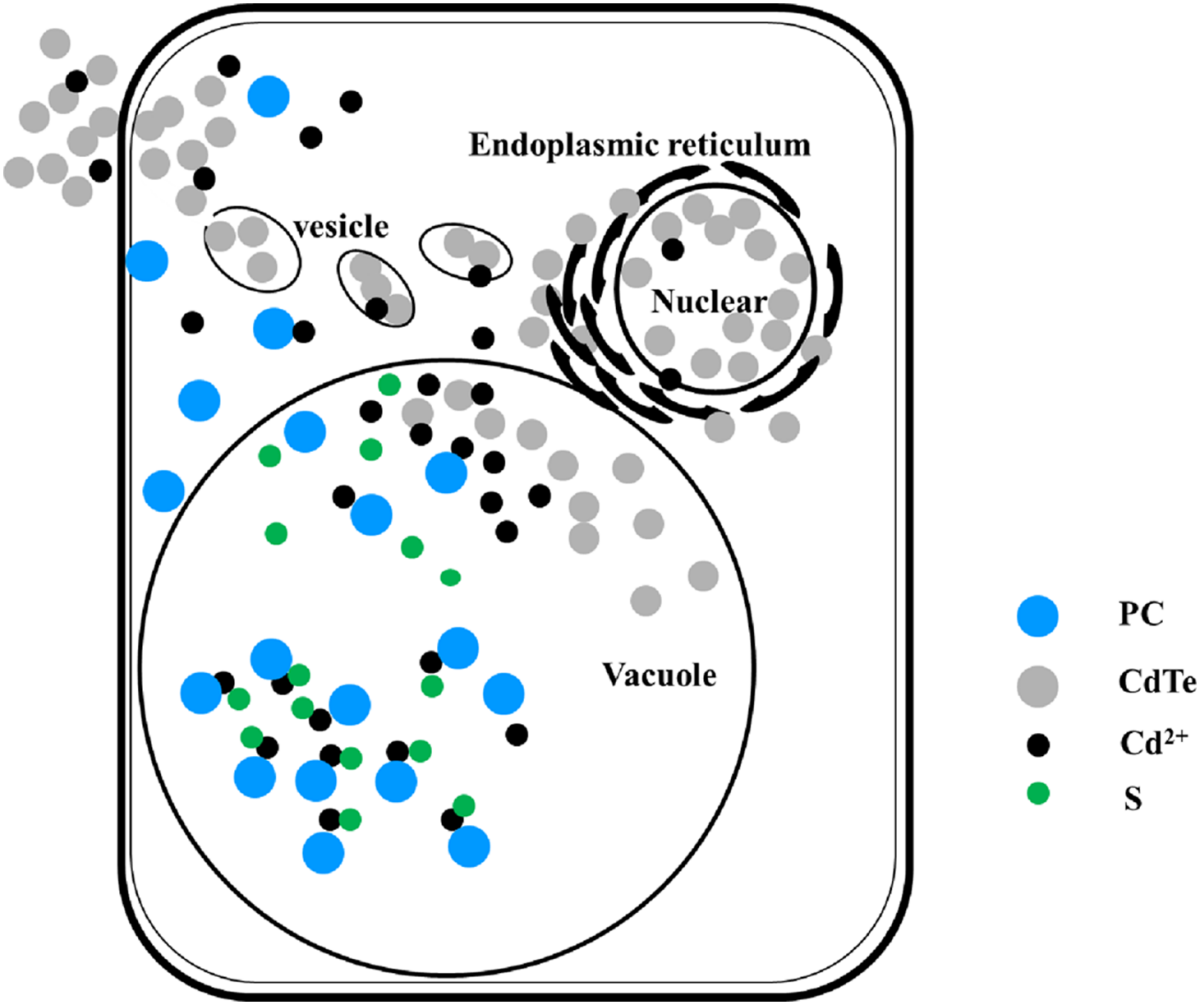
Schematic of a putative mechanism through which CdTe-QDs are processed by plant cells.

The presence of intracellular CdTe-QDs (g) was clearly visible after 3 days of CdTe-QDs treatment (g, j). Cell morphology appeared normal, with smooth surface and intact cell walls. After 4 days of treatment (h, k), vacuolation of cytoplasm appeared in some cells as shown ringed in red (k) and at higher magnification (s, t, u). Many small vesicles appeared around the nucleus (t), and CdTe-QDs were located inside the nucleus (u). This indicated the CdTe-QDs may enter the nucleus through the nuclear pore. CdTe-QDs were not uniformly distributed, but were found preferentially in and around the nucleus (u). A putative vesicle transport mechanism is shown in Figure 14. We propose that following the uptake of QDs by plant cells, PCs bind to Cd and form stable metal chelate complexes that are stored in the vacuole. Unbound excess QDs could spread into the nucleus leading to DNA damage. We inferred that NPs could produce a nanostructure that lead to an increase in the concentration of Cd around the nucleus, resulting in an increased cytotoxicity [12]. After 5 days of treatment (i, l), cell walls appeared looser and cells more variable in size (l), and the CdTe-QDs fluorescence intensity was reduced (i). The morphological characters resemble the features of programmed cell death.

In the combined UV-B and CdTe-QDs treated group, the situation is similar to the CdTe-QDs treated group. Cells exhibited normal morphology (p) on the third day and filled with CdTe-QDs (m). After 4 day treatment, cells are varied in size (n, q, white arrows). While following 5 day treatment, cells lost their square shape (r) and the fluorescence of CdTe-QDs was reduced dramatically (o).

**Figure 15:**
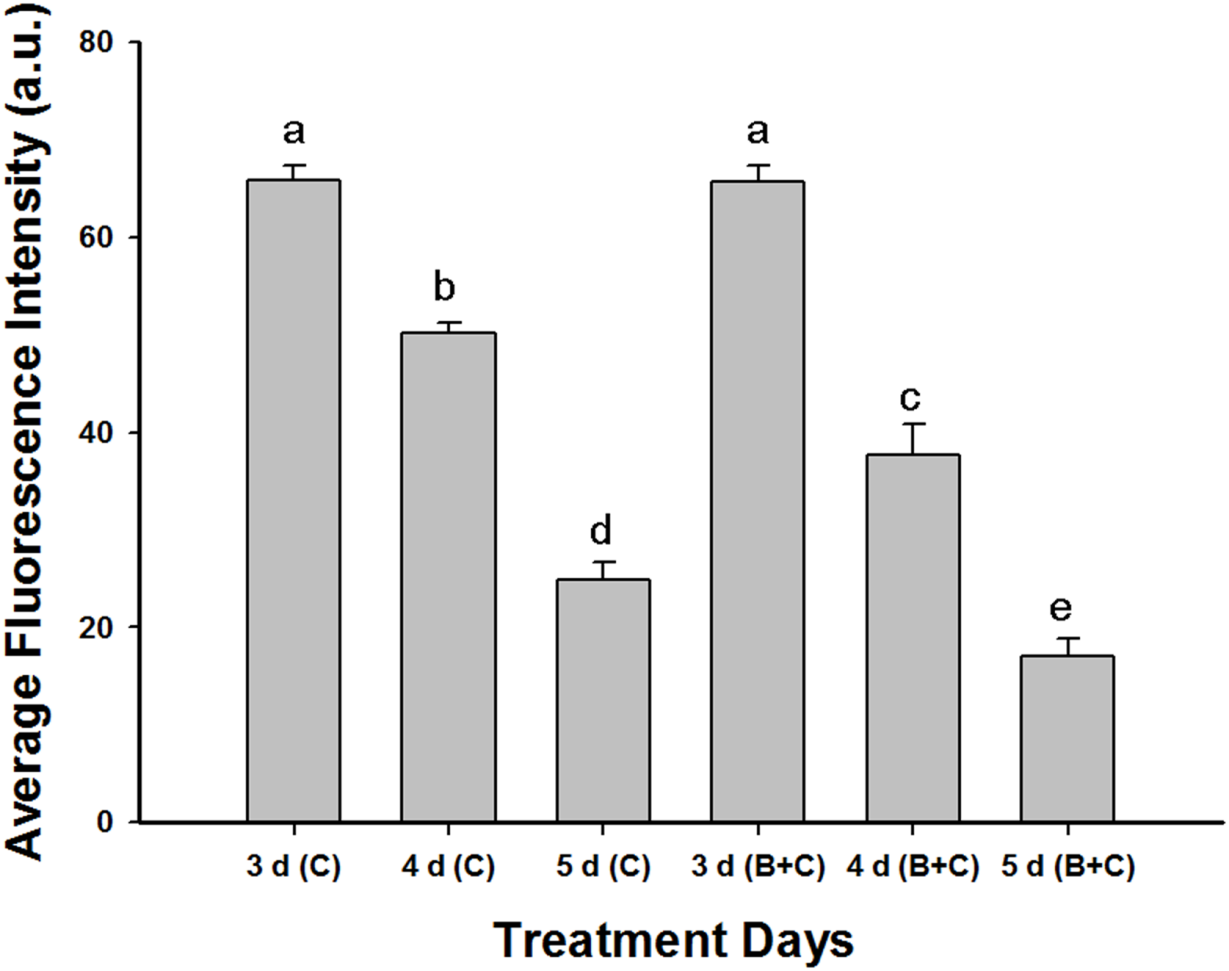
Average fluorescence intensity of QDs in 50 cells after different treatment durations. C, CdTe-QDs treatment alone; B+C, combined enhanced UV-B and CdTe-QDs treatment. Values are means±SD (n = 3). Means with the same letter are not significantly different at Tukey’s test (p≤0.05).

Fifty cells were subsequently selected and the change in the fluorescence intensity over the treatment period was measured (Figure 15). CdTe-QD-derived fluorescence decreased by ∼62.5% from the third to the fifth days of CdTe-QDs treatment and the decrease in intensity up to ∼74.0% was observed in the combined treatment group. The results showed the UV-B radiation accelerated the CdTe-QDs-derived fluorescence decay, that is, ∼11.5% fluorescence intensity decreased following the addition of UV-B radiation compared to single CdTe-QDs treatment. This was an order of magnitude higher than the inherent decrease in QDs emission intensity over 7 days (6.5%; Figure 3). The result indicated that CdTe-QDs were degraded by plant cells.

We detected the presence of QDs in shoots of seedlings but no fluorescence signal was observed in shoots. This suggested two possibilities. First, QDs may not have been transported to the top of the seedlings because QDs were only taken up by root epidermal cells and were not taken up by vascular tissues. Second, QDs that were taken up by root cells may have been degraded by PCs and stored in vacuoles, substantially decreasing the number of QDs were not available for transport into the shoot. Root cell took up the QDs and degraded them into Cd^2+^ partly. Cd^2+^ would bind to PCs or GSH, this is the cytotoxicity. While the other QDs changed ROS level and activated caspase, this would result in programmed cell death.

### Programmed cell death in seedling cells

**Figure 16:**
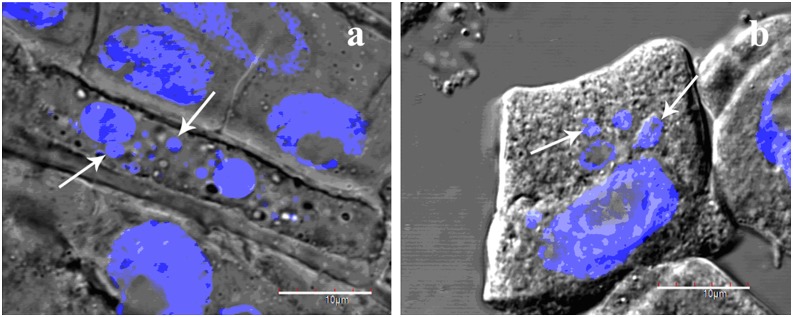
Cells undergoing programmed cell death. DAPI-stained DNA is shown in blue. Apoptotic bodies are indicated with white arrows. Shoot cells (a) and root cells (b) are shown. Scale bars = 10 µm.

Apoptotic bodies visualized by DAPI staining were found in root and stem cells of all three treatment groups while absence in the control group (Figure 16), suggesting an induction of programmed cell death following the treatments.

DNA laddering or fragmentation is a feature of apoptosis [51]. Programmed cell death was investigated by using DNA ladder analysis (Figure 12). DNA fragmentation visualized as DNA laddering was evident in root cells of treatment groups involving CdTe-QDs. This may be due to nucleic acid damage caused by CdTe-QDs presence in the nuclei. Free Cd^2+^ would lead to further DNA damage [52]. As oxidation of aromatic DNA bases is the main source of DNA damage, an increase in intracellular ROS levels induced by CdTe-QDs would further induce DNA damage, leading to enhanced cytotoxicity. At the cellular level, Cd^2+^ alone could inhibit the biosynthesis of DNA, RNA and protein, and induce DNA strand breakage and lipid peroxidation [52]. In the combined treatment, Cd^2+^ release would be enhanced by UV-B radiation, which would further enhance the cytotoxicity.

DNA fragementation in shoots was detected in the treatment groups involcing UV-B radiation, with the combined treatments producing the most DNA laddering. UV-B radiation causes DNA damage [53] through various mechanisms including formation of cyclobutyl pyrimidine dimer (CPD) adducts, (6-4) photoproducts ((6-4) PPs), inter/intra cross links (ICLs), 8-oxoguanine, and DNA double-strand breaks (DSBs) [48]. We inferred that the damage caused by enhanced UV-B exposure in shoots masked the Cd^2+^ damage effects.

In summary, QDs are widely used and are becoming increasingly dispersed in the environment. Our study revealed the cytotoxicity of CdTe-QDs and UV-B radiation on wheat seedlings. Our results demonstrated that CdTe-QDs nanoparticles and UV-B radiation affected the antioxidant defense system and programmed cell death in the roots and shoots of wheat seedlings. At the doses used (200 mg CdTe-QDs/L and 10.0 KJ/m^2^/d enhanced UV-B), treatments induced negative effects on antioxidant enzyme activities and resulted in the formation of apoptotic bodies and DNA fragmentation. Combined treatments with both CdTe-QDs and enhanced UV-B exhibited the added deleterious effects. It is currently impossible to distinguish between Cd released from industrial waste and those released from QDs. The precise concentrations of Cd emissions from QDs are thus unknown. Therefore, in vitro studies such as ours are necessary for generating mechanistic insights into the specific effect of CdTe-QDs on agricultural plants.
